# Mental Health Treatment in Adults with Congenital Heart Disease in Germany: An Online, Cross-Sectional Study of Status, Needs, and Treatment Reasons

**DOI:** 10.3390/jcdd12060231

**Published:** 2025-06-18

**Authors:** Anna-Lena Ehmann, Emily Schütte, Janina Semmler, Felix Berger, Ulrike M. M. Bauer, Katharina Schmitt, Constanze Pfitzer, Paul C. Helm

**Affiliations:** 1 Department of Congenital Heart Disease—Pediatric Cardiology, Deutsches Herzzentrum der Charité, Augustenburger Platz 1, 13353 Berlin, Germany; anna-lena.ehmann@charite.de (A.-L.E.);; 2 Charité—Universitätsmedizin Berlin, Corporate Member of Freie Universität Berlin and Humboldt-Universität zu Berlin, Charitéplatz 1, 10117 Berlin, Germany; 3 National Register for Congenital Heart Defects, Augustenburger Platz 1, 13353 Berlin, Germany; 4 Department of Obstetrics, Charité-Universitätsmedizin Berlin, Corporate Member of Freie Universität Berlin and Humboldt-Universität zu Berlin, Augustenburger Platz 1, 13353 Berlin, Germany; 5 Competence Network for Congenital Heart Defects, Augustenburger Platz 1, 13353 Berlin, Germany; 6 Department of Psycho-Cardiology, Deutsches Herzzentrum der Charité, Augustenburger Platz 1, 13353 Berlin, Germany

**Keywords:** congenital heart defect, mental health, psychotherapy, treatment, ACHD

## Abstract

Improved medical treatments have extended survival and life expectancy in adults with congenital heart defects (ACHD), placing greater emphasis on psychosocial health. Up to one-third of ACHD experience anxiety or depression, and half develop a mental illness during their lifetime. While there is solid evidence on the prevalence of mental health, many do not receive psychological, psychotherapeutic, or psychiatric treatment (PST) and the psychological care situation remains understudied. In a nationwide, online cross-sectional survey conducted in Q1 2024, 1486 ACHD aged 18 to 85 (M_age_ = 36.84 years; 60.8% female) registered in the German National Register for Congenital Heart Defects (NRCHD) completed self-report questionnaires on sociodemographics, illness identity (Illness Identity Questionnaire), mental well-being, and utilisation of PST. CHD diagnoses were determined in conformity with the International Pediatric and Congenital Cardiac Code (IPCCC) and CHD was classified according to Warnes et al. (simple/moderate/complex). Analyses included chi-square tests, t-tests, and binary logistic regression. Overall, 32.8% of participants reported current and/or previous PST (women 37.5%, men 25.3%). PST utilisation was significantly higher in those with complex (40.2%) compared to moderate (29.6%) and simple CHD (25.3%) (*p*s < 0.01). Primary treatment reasons were mental illness (41.7%) and CHD-related concerns (37.2%). Nearly half of treatments were self-initiated (45.8%) and about one-third were physician-recommended (30.8%). Logistic regression revealed CHD severity as a significant predictor of PST use (*p*s < 0.05), with lower odds for simple (OR = 0.48) and moderate (OR = 0.66) compared to complex CHD when controlling for sex (*p* < 0.001, OR = 1.87), age (*p* = 0.022, OR = 1.011), education level (*p*s between 0.060 and 0.780), and net income (*p*s < 0.05). Those receiving PST showed significantly higher maladaptive illness-identity scores (engulfment, rejection) and lower acceptance. Approximately one in three ACHD requires mental health support, particularly those with complex CHD. The CHD itself acts as a key stressor and treatment motivator. Findings underscore the need for integrated care linking cardiological and psychosocial services. Routine screening for psychological distress and low-threshold access to PST—also for patients with simple and moderate CHD—are essential to identify and address mental health needs early.

## 1. Introduction

Congenital heart defects (CHD) occur in about 0.8–1.2% of all live births [1]. Due to significant advancements in CHD treatment, survival rates and life expectancy have greatly improved [2,3,4]. As a result, psychosocial factors, including quality of life (QoL) and mental health, have become increasingly important [5,6].

CHD is associated with numerous everyday limitations and stressors, such as reduced physical performance, concerns about disease progression, social comparisons, or issues relating to career choice and family planning [7,8,9]. Numerous studies provide evidence that these illness-related stressors are associated with an increased risk of mental stress and illness in adults with CHD (ACHD). In particular, anxiety disorders and depression are common in this patient group. According to studies, around one-fourth to one-third of ACHD suffer from an anxiety disorder and/or depression [6,10,11,12,13,14], resulting in a lifetime prevalence of about 50% [12,15]. This increased risk of mental illness in CHD relates not only to anxiety disorders and depression, but is also evident in relation to bipolar disorders, psychosis, attention deficit hyperactivity disorder, post-traumatic stress disorder, personality disorder, and autism [6,12,16,17,18]. Results from German samples mainly suggest that a higher expression of depression and anxiety symptoms appears to exist regardless of CHD severity [19,20]. However, the relationship between CHD severity and mental health seems complex and multifaceted, and our study focuses on the utilisation of mental health treatment rather than the prevalence of mental health issues.

As a relevant factor in the context of mental distress in ACHD, illness identity, which evaluates the extent to which a chronic illness becomes part of one’s identity and influences self-perception, was identified in previous studies [21,22], potentially providing a starting point for explaining emotional stress in this patient group. While adaptive dimensions of illness identity such as acceptance and enrichment tended to be negatively correlated with symptoms of depression and anxiety, maladaptive dimensions such as engulfment and rejection tended to be associated with increased psychopathological symptoms. The emotional confrontation with the illness itself therefore plays a central role in the experience of emotional stress and mental illness and is discussed as a moderating factor [21].

Mental health itself was identified as a predictor of quality of life in ACHD [6,10] and is also linked to perceived state of health and exercise capacity [14,23], which makes it particularly important to consider psychological well-being for a comprehensive care of the patient group.

Although it is known that chronic illnesses in general are associated with a higher risk of mental illness [24], mental stress in ACHD has so far been insufficiently recognised and treated [10,12,25]. In their review, Kovacs et al. [8] emphasise that mental health treatment is still uncommon despite improved medical care. Overall, research on the treatment of mental health issues in ACHD is still limited and robust data on this clinically highly relevant topic are missing [26].

Therefore, this study addresses the aforementioned research gap and records the utilisation of psychological, psychotherapeutic, and psychiatric treatment (PST) in ACHD in Germany. The aim of this study is to identify the reasons for treatment as well as possible sex and CHD severity differences in addition to the utilisation of the corresponding treatment. To the best of our knowledge, this is the first nationwide study to investigate the use of mental health treatment services in this patient group.

## 2. Materials and Methods

### 2.1. Study Design

This online-based cross-sectional study examined PST use and illness identity in ACHD. Data collection was conducted during the first quarter of 2024. A total of 1486 ACHD patients from the National Register for Congenital Heart Defects (NRCHD) participated by completing self-report questionnaires on sociodemographic data, illness perception and identity, emotion regulation, mental well-being, and the utilisation of cardiological and psychological treatment. Participants were invited via email, and their medical records from the NRCHD database were included in the statistical analysis. Cardiac conditions were classified using the internationally recognised Warnes et al. [27] severity classification and the International Pediatric and Congenital Cardiac Code (IPCCC) [28]. The Warnes et al. classification system categorises CHD into simple, moderate, and complex based on anatomical complexity, clinical course, and care needs. Simple defects have favourable outcomes and minimal follow-up, moderate ones require interventions and regular monitoring, while complex cases involve complicated anomalies needing lifelong specialised care. This widely used system guides clinical management, research, and resource planning [27].

### 2.2. National Register for Congenital Heart Defects

The NRCHD is located in Berlin, Germany, and holds medical records for around 60,000 individuals with CHD (as of March 2025), making it one of the most extensive CHD databases in Europe. Serving as a key resource for clinical research in this field, the NRCHD facilitates studies on various aspects of CHD [23]. Participation is voluntary and requires patients to provide general consent, allowing the NRCHD to gather and store medical data from their treating physicians. This consent applies to both ongoing and future research, while participants retain the right to revoke it at any time.

### 2.3. Measures

#### 2.3.1. Mental Health Treatment

Patients were asked about their current or past involvement in PST, as well as whether they were previously or at the time of the survey undergoing such treatments. They were also asked if they were on a waiting list for psychotherapy. Additionally, multiple reasons for seeking treatment could be selected, and the person who initiated the treatment, as well as the healthcare professional providing it, were recorded. As studies indicate a gap between prevalence and treatment [12,15,25], the selected questions aimed to identify potential barriers and care pathways but also to explain the decision and motivation to start treatment.

#### 2.3.2. Illness Identity

The Illness Identity Questionnaire (IIQ) [22] assesses the extent to which individuals incorporate their illness into their personal identity. This standardised and validated questionnaire has already been used several times in ACHD samples, has been associated with healthcare use and has demonstrated good reliability in this population [21,26,29,30,31]. It comprises four distinct dimensions: engulfment and rejection, which reflect a lack of integration, and acceptance and enrichment, which represent more adaptive ways of integrating the illness into one’s identity. Each dimension is evaluated using multiple items, rated on a five-point scale ranging from 1 (lowest level) to 5 (highest level). Mean scores are calculated for each dimension. Cronbach’s alpha was calculated to determine the internal consistency. This was 0.92 for the engulfment scale (8 items), 0.81 for rejection (5 items) and acceptance (5 items), 0.94 for the enrichment scale (7 items), and 0.83 for the whole IIQ (25 items).

### 2.4. Statistical Analyses

Data were analysed using SPSS (Version 29.0). We performed chi-square tests to compare group differences by sex and CHD severity. Binary logistic regression was conducted with treatment use (past and/or current vs. never) as a dependent variable and CHD severity (simple/moderate/complex), age, sex, education level, and net income (categorised into low: <1750 €, medium: 1750 €–3999 € and high: >3999 €) as independent variables to identify predictors of PST use. As 127 people did not provide any information on their net income, and 223 patients were excluded from the analyses due to lack of medical information concerning CHD severity (‘no class’), the logistic regression was carried out with a reduced sample size of N = 1136. The final sample remained sufficiently large to ensure statistical power. A *t*-test for independent samples was conducted to evaluate significant differences in illness identity dimensions between ACHD who received vs. not received PST at the time of the survey. The significance level was set at 0.05.

### 2.5. Ethical Statement

The Charité granted an ethical approval for this study (EA4/178/22). Participants provided written informed consent to take part in this study.

## 3. Results

### 3.1. Study Cohort

Overall, 1486 ACHD (M_age_ = 36.8 years, SD_age_ = 14.6; 60.8% female) took part in the survey and were classified by CHD severity according to Warnes et al. [27] into simple (n = 162, 10.9%), moderate (n = 686, 46.2%), and complex (n = 415, 27.9%). For a further 223 patients (15.0%), insufficient medical data were available for classification, but these were nevertheless included in analyses in which no distinction was made between severity levels. Table 1 summarises the sociodemographic characteristics of the overall sample and each CHD severity group.

### 3.2. Use of Psychological, Psychotherapeutic, or Psychiatric Treatment

A total of 32.8% stated that they are currently undergoing and/or have previously undergone PST. However, 67.2% of respondents stated that they had never received such treatment. Treatment was used more frequently by women than by men (37.5% vs. 25.3%). At the time of the survey, 5.1% were on a waiting list for psychotherapy.

#### 3.2.1. Comparison Between CHD Severity Levels

**Figure 1:**
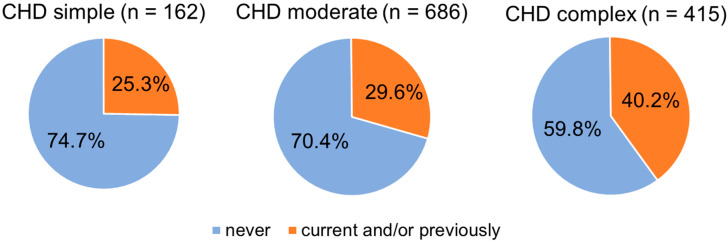
Use of psychological, psychotherapeutic, or psychiatric treatment (PST) depending on CHD severity. For 223 patients, insufficient medical data were available for CHD classification (‘no class’), of which 34.1% were undergoing PST current and/or previously.

Of the patients with simple CHD, 25.3% stated that they had received appropriate treatment at the time of the survey and/or previously, compared with 29.6% of patients with moderate CHD and 40.2% of those with complex CHD. Chi-square tests revealed a significant difference between simple and complex (*p* = 0.009) and moderate and complex (*p* = 0.003) CHD severity, while there was no significant difference between simple and moderate (*p* = 0.747) CHD severity regarding treatment use. Figure 1 displays the use of mental health treatment depending on CHD severity.

#### 3.2.2. Treatment Reasons

**Figure 2:**
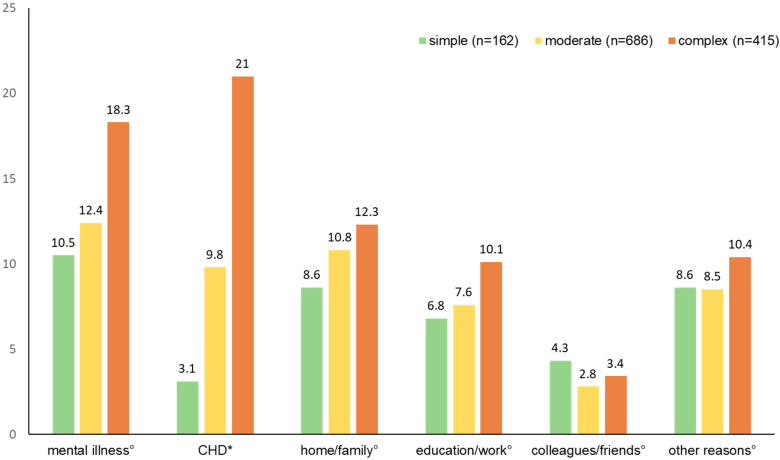
Reasons for psychological, psychotherapeutic, or psychiatric treatment (PST) depending on CHD severity. Multiple answers were possible, so percentages are not mutually exclusive. Chi-square tests were conducted for group comparisons between CHD severity levels. ° *p* > 0.05, * *p* < 0.001. For 223 patients, insufficient medical data were available for CHD classification (‘no class’). This subgroup was not included in the statistical analyses for this illustration.

A total of 41.7% reported mental illness as the reason for undergoing PST, while 37.2% attributed their treatment to CHD. Additionally, 32.6% mentioned home or family issues, 25.3% pointed to education or work concerns, and 9.2% referred to influences from colleagues or friends. Furthermore, 28.1% indicated reasons beyond these categories. Multiple answers were possible. Figure 2 illustrates the treatment reasons for each CHD severity level.

#### 3.2.3. Treatment Initiation

In total, 45.8% of respondents initiated treatment independently. In 30.8% of cases, treatment was initiated by a doctor and in 19.9% of cases, friends or family were the initiator. In 3.5% of cases, treatment was initiated by another person or institution.

#### 3.2.4. Practitioner

Overall, 78.6% were treated by a psychologist or psychotherapist. In 15.6% of cases, treatment was provided by a doctor. In 5.7% of cases, treatment was carried out by a person with other qualifications.

#### 3.2.5. Mental Health Treatment and Illness Identity

ACHD who were receiving PST at the time of the survey reported a significantly worse illness identity in the dimensions of engulfment (*p* < 0.001, 95%-CI: [−0.71, −0.45]), rejection (*p* < 0.001, 95%-CI: [−0.37, −0.14]), and acceptance (*p* < 0.001, 95%-CI: [0.20, 0.40]) compared to those who were not in treatment. There were no significant differences in the dimension of enrichment (*p* = 0.431, 95%-CI: [−0.09, 0.21]). Table 2 displays the descriptive values of illness identity for the two subgroups.

#### 3.2.6. Predictors of PST

Binary logistic regression revealed CHD severity as a further significant factor (reference complex: *p* < 0.001, simple: *p* = 0.001, B = −0.740, OR = 0.477, 95%-CI: [0.307, 0.742], moderate: *p* = 0.003, B = −0.419, OR = 0.658, 95%-CI: [0.498, 0.869] in predicting the use of PST when controlling for sex (reference male: *p* < 0.001, B = 0.624, OR = 1.867, 95%-CI: [1.422, 2.451]), age (*p* = 0.022, B = 0.011, OR = 1.011, 95%-CI: [1.002, 1.021]), education level (reference university: all *p*s > 0.05), and net income (reference low: *p* = 0.003, medium: *p* = 0.012, B = −0.376, OR = 0.687, 95%-CI: [0.512, 0.922]; high: *p* = 0.002, B = −0.770, OR = 0.463, 95%-CI: [0.285, 0.750]). Nagelkerkes R^2^ was 0.073. The results of the binary logistic regression can be found in Table 3.

## 4. Discussion

This study was the first to investigate the utilisation of mental health treatment for ACHD nationwide in Germany, whereas previous studies have mainly focussed on the prevalence of mental illnesses. Our results provide evidence that a significant proportion of ACHD experience mental distress. Approximately one-third are currently and/or previously receiving treatment.

Our study suggests that a more complex CHD is associated with increased utilisation of mental health treatment. CHD severity was a significant predictor in the binary logistic regression when controlling for sociodemographic factors, indicating that a more complex CHD was associated with a higher chance of PST use current and/or in the past. Among the socio-demographic factors, being female, higher age, and lower net income showed a significant association with higher PST use. However, education level was insignificant.

Previous results questioning the relationship between medical aspects and mental health are controversial. Studies with German samples mainly report no significant association between CHD severity and anxiety or depression symptoms [19,20]. Some other trials with international samples report correlations between symptoms of depression and anxiety and CHD complexity, which are usually inferior in multivariate analyses due to the effect of the NYHA class [14,32,33,34]. Considering the functional status, studies only partially report a significant correlation between NYHA class and mood or anxiety disorders [6,15]. For other mental illnesses such as personality disorders, the NYHA class was found to be insignificant [18]. Moreover, in their review, Moons et al. [6] report no evidence for a significant influence of the NYHA class in the context of eating disorders, post-traumatic stress disorders, or schizophrenia. In addition, the methodological validity of the measurement of functional status with the NYHA class has been criticised in some studies, as it is considered subjective and has poor interrater reproducibility [35,36,37]. In view of the study situation, neither the relationship between CHD severity nor the NYHA class therefore appears to be robust across all mental illnesses. Although the literature, especially German samples, does not predominantly indicate a strong association between CHD complexity and depression or anxiety, our results show differences in the use of PST depending on CHD severity. In conjunction with previous studies, our results therefore suggest that the utilisation of mental health treatment does not necessarily correlate with the level of psychological distress but may be influenced by other factors such as better integration into the healthcare system and lower barriers to accessing treatment. Overall, the logistic regression model showed a rather small variance explanation, so that other potential predictors for the utilisation of PST, such as functional status, should be taken into account in further studies.

A potential hypothesis for the increased use of mental health treatment among people with complex CHD might be that this patient group is less afraid of stigmatisation because they are already confronted with a severe illness. Seeking help to maintain health may have become the norm for these individuals, reducing mental barriers associated with mental health treatments. As hypothesised above, people with complex CHD may be better integrated into the healthcare system, making it easier for them to access PST. This would emphasise the need to offer targeted psychosocial treatment to individuals with simple and moderate CHD and to make it easier for them to access psychotherapy. It should also be noted that we did not look at specific mental illnesses in this study, as it was the case in the previously mentioned studies about mental health in ACHD.

The CHD itself was the second most frequently cited reason for seeking treatment when looking at the overall sample. For those with complex CHD, the CHD was the most frequently cited reason, ahead of mental illness itself. In comparison, individuals with simple and moderate CHD severity cited the CHD itself as the second most common reason for treatment, while mental illness was stated as the most common treatment reason. Considering the concept of illness identity, which describes how strongly individuals identify with their chronic illness and what role it plays in their self-image, it could be assumed that individuals with complex CHD identify more strongly with their illness, compared to patients with simple or moderate complexity. This hypothesis is supported by a study highlighting that people with complex CHD feel more engulfed by the CHD than those with lower disease complexity [31]. This, in turn, could lead to the psychological burden being attributed more to CHD, both by those affected themselves and possibly by the environment or medical professionals. As discussed above, the concept of stigmatisation might also play a central role in this context. As patients with less complex CHD are generally less integrated into the medical system, there may be a higher threshold for discussing psychological problems in connection with the physical illness. As a result, psychological and somatic challenges may be viewed as separate issues.

Although there are studies which show that the QoL of CHD patients is sometimes as good or better than that of healthy control subjects [5,38], our findings suggest that the CHD itself can nevertheless be a significant burden in the lives of those affected. This underscores that the emotional distress associated with CHD is of great importance and should definitely be taken into account for comprehensive treatment.

Overall, the literature to date calls for further research and longitudinal studies to better understand the exact cognitive and emotional mechanisms behind this. One starting point is the finding that QoL is rated as good or better than that of healthy controls when it comes to life satisfaction per se. When QoL is measured as physical functioning, the more complex the CHD, the worse CHD patients describe their QoL [5]. If the functional status is taken into account, from a comprehensive psychophysical perspective, it seems important to consider that the CHD could become a perpetuating factor for other psychological stress due to the limited physical resilience. The limited exercise capacity might consequently lead to increased avoidance and social withdrawal behaviour. Further longitudinal studies should follow in order to better explain the mechanisms of action.

Our results emphasise the important role of treating physicians in the context of mental health care. Over 30% of those surveyed stated that PST was initiated by their doctor. In just under half of the cases, treatment was initiated by the patients themselves. In order to guarantee a holistic treatment approach, we therefore also encourage the treating cardiologists and ACHD-physicians to directly address the psychological well-being of the patients and thus, initiate the potential utilisation of a support service.

ACHD who were undergoing PST at the time of the survey showed a more maladaptive illness identity (more rejection and engulfment, less acceptance). This could indicate that a problematic illness identity is associated with greater psychological stress, which may increase the need for psychotherapeutic/psychiatric support. These results are in line with previous findings [21]. Alternatively, it could also be that patients with greater psychological distress are more likely to develop a negative illness identity. As this is a cross-sectional study, no cause-and-effect relationship can be established and further studies are necessary. Nevertheless, our results confirm that illness identity should be taken into account in the context of mental illness and psychological distress in ACHD and should also play a central role in appropriate mental health treatment.

Although this study provides valuable insights into the utilisation of mental health treatment and mental stress experienced among ACHD in Germany, some limitations must be considered. First, the cross-sectional design of this study restricts the ability to draw causal inferences about the relationship between CHD severity and the use of PST. Longitudinal studies would be necessary to better understand how the utilisation of mental health care evolves over time in this patient group. Second, this study relied on self-reported data, which may be subject to recall bias or social desirability bias, as participants may not accurately report their treatment history or reasons for seeking care. Third, the study sample is derived from the NRCHD, which may limit generalisability to the broader ACHD population, particularly outside of Germany. Patients with insufficient information regarding CHD severity classification and net income were excluded from the relevant statistical analyses. Despite this exclusion, the final sample size remained sufficiently large to ensure analytical robustness. We note that future studies should incorporate alternative recruitment methods—such as telephone outreach or in-clinic enrolment—to ensure inclusion of harder-to-reach patients without reliable internet access. Moreover, prospective research across diverse healthcare settings is needed to assess the stability and wider applicability of our findings over time. Additionally, the absence of a control group, such as individuals without CHD, restricts our ability to compare mental health treatment utilisation between those with and without the CHD condition. As there was no direct patient contact in our study, we were unfortunately unable to include functional capacity, even though the literature indicates that there may be correlations here in the context of psychological stress and QoL. Finally, the data collection period was limited to the first quarter of 2024, and the findings may not reflect changes in treatment utilisation or mental health trends beyond that timeframe.

### Clinical Implications and Actionable Recommendations

For clinical practice, our results emphasise the need for increased interdisciplinary and multidisciplinary collaboration between cardiologists and psychologists, psychotherapists, or psychiatrists. The integration of psychosocial treatment services in ACHD-specialised centres could ideally lower the inhibition threshold for initiating and using mental health services. Specifically, our results further support the hypothesis that psychosocial counsellors working with this patient group should have a basic understanding and background knowledge of the chronic illness and CHD-related stressors in order to provide patients with the best possible support, also with regard to the concept of illness identity. The idea of psychological services integrated into cardiological treatment is also explicitly promoted in a meta-analysis on mental health interventions in ACHD [8].

In order to systematically consider psychosocial stress factors in the care of ACHD, routine screening for psychological distress and maladaptive illness identity should be performed. Validated short questionnaires such as the Patient Health Questionnaire-9 (PHQ-9; [39]) for recording depressive symptoms, the Generalised Anxiety Disorder Scale-7 (GAD-7; [40]) for recording anxiety symptoms, and the Illness Identity Questionnaire (IIQ; [22]) for assessing individual disease management offer a practicable option for the standardised recording of relevant psychosocial parameters in everyday clinical practice [20]. The integration of these instruments into ACHD outpatient clinics—ideally at defined points in time such as initial presentation, follow-up appointments, or in the event of clinical deterioration—makes it possible to identify vulnerable patients at an early stage. The data collected can be used specifically to determine the indications for further psychocardiological or psychosocial interventions and thus, contribute to holistic, patient-centred care. In addition, medical and nursing staff should be trained in the use of the instruments and in communicating the sensitive results in order to ensure adequate interpretation and therapeutic utilisation of the screening findings.

To reduce barriers, cardiologists should routinely ask their patients proactively about their mental state. Psychoeducational information from cardiologists about the increased risk of suffering from a mental illness with CHD might also counteract stigmatisation and simplify the first step towards psychological treatment. Ideally, doctors should have information material on psychosocial treatment programs which can be handed out if there are signs of psychological burden. Additionally, our findings suggest that individuals with more complex CHD may be more likely to seek mental health treatment due to better integration into the healthcare system, highlighting the importance of making mental health services accessible to all ACHD patients, regardless of disease severity.

For the specific psychological treatment of mental disorders in chronic illnesses, some studies suggest the approach of cognitive behavioural therapy (CBT). By adapting CBT to chronic illnesses, several goals can be pursued: in addition to providing psychoeducation on the link between chronic illness and mental stress, therapy can focus on restructuring illness-related cognitions, developing strategies for rest and recovery management, and strengthening personal resources [41,42]. Although studies on psychotherapeutic interventions for ACHD are still sparse [43,44] and the concept of illness identity is not yet addressed in this context, results from the U.S., for example, underscore that cognitive therapy, as well as skills training, can reduce symptoms of emotional distress in ACHD [45]. The concept of the ‘sense of coherence’ (SOC), originating from positive psychology approaches and describing the feeling that life is understandable, manageable and meaningful, has also already been introduced as a potential therapeutic starting point [6]. The SOC has already been linked to perceived health and quality of life in people with CHD, and is seen as a protective factor which may therefore play an important role in the context of illness identity and coping with the disease [46,47,48,49].

## 5. Conclusions

This study provides important evidence on the utilisation of mental health treatment among ACHD in Germany and comprises a large sample. It highlights the significant mental health challenges faced by ACHD patients, with approximately one-third of the participants reporting current and/or previous psychological treatment. The findings indicate that the severity of CHD is a factor influencing the likelihood of receiving mental health care, with individuals with more complex CHD being more likely to seek treatment, also when controlling for sociodemographic characteristics. Furthermore, the emotional distress associated with CHD itself is reported as a key reason for seeking psychological support, emphasising the need for a comprehensive approach to care that addresses both the physical and psychological well-being of ACHD patients. Additionally, our results indicate that illness identity is associated with psychological distress in ACHD, providing a potential starting point for tailored interventions. Our results underscore the importance of integrating mental health care into the clinical management of ACHD, especially in order to also include patients with simple and moderate CHD, and call for increased awareness and accessibility of psychological support services for this vulnerable patient group.

## Figures and Tables

**Table 1:** Demographic characteristics of the total sample and by CHD severity level.

Demographic Data	Total Sample (N = 1486)	CHD Simple (n = 162)	CHD Moderate (n = 686)	CHD Complex (n = 415)	CHD No Class (n = 223)
Age in years, M (SD)	36.8 (14.6)	33.2 (15.0)	38.2 (14.6)	34.1 (12.8)	40.3 (15.9)
Gender, n (%)					
Male	580 (39.0)	47 (29.0)	291 (42.4)	163 (39.3)	79 (35.4)
Female	903 (60.8)	115 (71.0)	395 (57.6)	249 (60.0)	144 (64.6)
Non-Binary	3 (0.2)	0 (0)	0 (0)	3 (0.7)	0 (0)
Relationship status, n (%)					
Single	542 (36.5)	59 (36.4)	228 (33.2)	180 (43.4)	75 (33.6)
In a relationship/married	944 (63.5)	103 (63.6)	458 (66.8)	235 (56.6)	148 (66.4)
Number of school years, M (SD)	11.40 (1.53)	11.44 (1.34)	11.39 (1.51)	11.35 (1.69)	11.47 (1.43)
Education, n (%)					
Without degree	36 (2.4)	4 (2.5)	16 (2.3)	13 (3.1)	3 (1.3)
Pupil	42 (2.8)	5 (3.1)	19 (2.8)	14 (3.4)	4 (1.8)
Elementary school (9 years)	68 (4.6)	2 (1.2)	31 (4.5)	25 (6.0)	10 (4.5)
Secondary school (10 years)	147 (9.9)	18 (11.1)	60 (8.7)	43 (10.4)	26 (11.7)
Completed apprenticeship	297 (20.0)	23 (14.2)	144 (21.0)	87 (21.0)	43 (19.3)
Advanced technical college	148 (10.0)	23 (14.2)	63 (9.2)	39 (9.4)	23 (10.3)
High school diploma	247 (16.6)	28 (17.3)	115 (16.8)	58 (14.0)	46 (20.6)
University	501 (33.7)	59 (36.4)	238 (34.7)	136 (32.8)	68 (30.5)
Employment, n (%)					
In education	310 (20.9)	50 (30.9)	130 (19.0)	88 (21.2)	41 (18.4)
Working full-time	601 (40.4)	63 (38.9)	303 (44.2)	153 (36.9)	82 (36.8)
Working part-time	328 (22.1)	34 (21.0)	138 (20.1)	103 (24.8)	53 (23.8)
Self-employed	77 (5.2)	7 (4.3)	39 (5.7)	18 (4.3)	13 (5.8)
Jobseeker	26 (1.7)	3 (1.9)	11 (1.6)	7 (1.7)	5 (2.2)
Retired	179 (12.0)	10 (6.2)	80 (11.7)	54 (13.0)	35 (15.7)
Other	84 (5.7)	7 (4.3)	36 (5.2)	32 (7.7)	9 (4.0)
Net income, n (%)					
<1250 €	335 (22.5)	37 (22.8)	142 (20.7)	106 (25.5)	50 (22.4)
1250–1749 €	188 (12.7)	19 (11.7)	83 (12.1)	56 (13.5)	30 (13.5)
1750–2249 €	207 (13.9)	22 (13.6)	98 (14.3)	61 (14.7)	26 (11.7)
2250–2999 €	278 (18.7)	34 (21.0)	131 (19.1)	67 (16.1)	46 (20.6)
3000–3999 €	162 (10.9)	21 (13.0)	76 (11.1)	45 (10.8)	20 (9.0)
4000–4999 €	74 (5.0)	3 (1.9)	36 (5.2)	23 (5.5)	12 (5.4)
≥5000 €	89 (6.0)	6 (3.7)	52 (7.6)	18 (4.3)	13 (5.8)
Not specified	153 (10.3)	20 (12.3)	68 (9.9)	39 (9.4)	26 (11.7)
PST *, n (%)					
In the past only	276 (18.6)	24 (14.8)	117 (17.1)	89 (21.4)	46 (20.6)
Current only	79 (5.3)	6 (3.7)	28 (4.1)	28 (6.7)	17 (7.6)
Past and current	132 (8.9)	11 (6.8)	58 (8.5)	50 (12.0)	13 (5.8)
No PST	999 (67.2)	121 (74.7)	483 (70.4)	248 (59.8)	147 (65.9)
Waiting for therapy place °	76 (5.1)	7 (4.3)	30 (4.4)	25 (6.0)	14 (6.3)

*Note*. Multiple answers were possible for employment. CHD = congenital heart defects. SD = standard deviation. PST = Psychological, psychotherapeutic, or psychiatric treatment. * Chi-square tests were performed for group comparison and revealed significant differences between CHD severity levels (simple/moderate/complex/no class) in PST use, *p* < 0.05. ° Chi-square test revealed no significant differences in waiting list status between CHD severity levels, *p* = 0.380.

**Table 2:** Descriptive statistics of illness identity dimensions by current PST use (yes vs. no).

Illness Identity Dimension	Use of PST at Time of Survey	Mean Value	Standard Deviation
Engulfment	No	1.93	0.78
	Yes	2.51	0.91
Rejection	No	1.99	0.90
	Yes	2.25	0.77
Acceptance	No	4.16	0.69
	Yes	3.86	0.69
Enrichment	No	3.23	1.05
	Yes	3.17	0.97

*Note.* N = 1486. PST = Psychological, psychotherapeutic, or psychiatric treatment. PST use ‘no’: n = 1275. PST ‘yes’: n = 211.

**Table 3:** Results of binary logistic regression predicting PST use (current and/or previous vs. never) based on CHD severity and sociodemographic characteristics.

Variables	B	SE	df	*p*	Exp(B)	95%-CI	
						Lower	Upper
Constant	−0.806	0.264	1	0.002	0.447		
CHD severity (Ref: complex)			2	<0.001			
Simple	−0.740	0.225	1	0.001	0.477	0.307	0.742
Moderate	−0.419	0.142	1	0.003	0.658	0.498	0.869
Sex (0 = male)	0.624	0.139	1	<0.001	1.867	1.422	2.451
Age	0.011	0.005	1	0.022	1.011	1.002	1.021
Education level (Ref: University)			7	0.496			
Without degree	−0.333	0.440	1	0.450	0.171	0.303	1.698
Pupil	−1.187	0.789	1	0.132	0.305	0.065	1.433
Elementary school (9 years)	0.091	0.327	1	0.780	1.096	0.577	2.079
Secondary school (10 years)	−0.234	0.246	1	0.342	0.791	0.488	1.283
Completed apprenticeship	−0.173	0.182	1	0.342	0.841	0.589	1.202
Advanced technical college	−0.72	0.232	1	0.755	0.930	0.590	1.466
High school diploma	−0.404	0.215	1	0.060	0.668	0.438	1.017
Net income (Ref: Low)			2	0.003			
Medium	−0.376	0.150	1	0.012	0.687	0.512	0.922
High	−0.770	0.247	1	0.002	0.463	0.285	0.750

*Note*. N = 1136. CHD = congenital heart defects. B = regression coefficient. SE = standard error. Df = degrees of freedom. *p* = *p*-value. Exp(B) = exponential B (equals odds ratio). CI = Confidence interval. CHD ‘no class’ (n = 223) were excluded from the logistic regression analysis.

## Data Availability

Data cannot be shared for data protection reasons.
